# Simulation and multi-objective optimization of the dimethyl carbonate production process

**DOI:** 10.1038/s41598-023-44100-y

**Published:** 2023-10-06

**Authors:** Ali Maleki, Fatemeh Bahadori

**Affiliations:** Faculty of Chemical Engineering, Urmia University of Technology, P.O. Box 57166-17165, Urmia, Iran

**Keywords:** Climate sciences, Chemical engineering

## Abstract

Greenhouse gases such as CO_2_ are considered effective materials in global warming due to their high absorptivity. So, lowering atmospheric CO_2_ is one of the most practical strategies. Utilizing carbon dioxide in chemical processes is an applicable method. In this study, Aspen HYSYS 10 was used to investigate how carbon dioxide can be added to the process of dimethyl carbonate production, and the affective parameters of the process, including temperature, residence time, feed ratio, and recycle ratio, were evaluated. It was observed that the production of DMC grew as temperature rose. The simulation results also revealed that a maximum conversion of roughly 8% was attained in the MeOH/EC. Additionally, boosting the recycle ratio is detrimental, and the impact of temperature and MeOH/EC has been enhanced by increasing the residence time. The interactions of the above parameters have been studied by Design Expert 12. The optimum value of effective parameters for the production of dimethyl carbonate has been obtained as follows: temperature of 164.7° C, recycle ratio of 0.2, residence time of 139.45 min, and feed ratio of 5.9%, leading to the conversion of 70%.

## Introduction

Carbon dioxide is one of the most important greenhouse gases resulting in climate change and global warming. CO_2_ has increased by more than 30% in the atmosphere since the industrial revolution in the seventeenth century. An increase in the earth's temperature due to the rise in CO_2_ emissions may lead to the melting of the polar ice caps, growth in forest fires, rising seas level, expansion of deserts, loss of animal habitat, spread of tropical diseases, and frequent and severe storms. Therefore, researchers are looking for solutions to reduce CO_2_ emissions^1^.

Conventional technologies for reducing the CO_2_ in the atmosphere are Enhanced Oil Recovery (EOR) by carbon dioxide injection, CO_2_ storage in depleted oil reservoirs, mineral carbonation, and recovery of CO before converting to CO_2_^2,3^. Another method is chemically converting CO_2_ into useful compounds as a valuable carbon source. However; synthesizing chemicals using CO_2_ as a raw material is still a developing issue because CO_2_ is not only thermodynamically stable but also kinetically neutral^4^. Currently, CO_2_ is implemented to produce methanol^5^, formic acid^6^, olefins^7^, urea^8,9^, and gasoline range hydrocarbons^10,11^. The mentioned processes almost require high energy for providing activation energy of reactions. In other words, a huge amount of CO_2_ is emitted during the combustion process to supply energy for a CO_2_-consuming reaction. So, it is necessary to develop CO_2_-consuming processes that occur in a low temperature conditions. One of the processes utilize CO_2_ in chemical reactions in low-temperature is alkyl carbonates production process.

Dialkyl carbonates are formed by binding two alkyl groups to CO. Dimethyl carbonate (DMC) is the smallest alkyl carbonate, consumed globally by more than 90,000 ton/yr, mainly as an intermediate compound in polycarbonate production, solvent, a substitute for toxic agents, such as phosgene and methylated groups like methyl sulfate and methyl chloride. In addition; DMC, as an oxygenated additive, is a suitable alternative to dimethyl tert-butyl ether and reduces the emission of pollutants during the combustion of fuels^12,13^. DMC can be produced from phosgene through partial carbonylation of methanol (Bayer process) and methylene nitrile (Obi process). Due to poisonous nature of phosgene and methylene nitrile, the alternative processes based on direct and indirect carbonylation have been developed [1, 12, and 14].

New alternative processes for the production of DMC include the transesterification of ethylene carbonate, transesterification of urea, and direct production from CO_2_ and alcohol as raw materials^1,12^.

Producing DMC from alcohols, obtained from renewable sources, and CO_2_, a greenhouse gas, is highly attractive due to the raw materials. One of the important processes for the effective use of CO_2_ is the production of Ethylene Carbonate (EC) by cyclization of CO_2_ with Ethylene Oxide (EO) and consequently reaction of produced ethylene carbonate with methanol to produce DMC. As an environment-friendly process, this process utilizes CO_2_ and methanol more conveniently than other processes. In addition; during the process, precious by-products of ethylene carbonate, ethylene glycol, and 2-Methoxyethanol are produced. So, it is necessary to evaluate and improve the process for developing it for further applications. A simulation study provides a better and more accurate combination of the parameters and factors affecting the process. The separation unit for the process has been simulated and analyzed by Souza et al.^15^ and in this study; the reaction unit has been simulated and optimized to study the process profoundly and determine the optimal condition of the reaction.

## Simulation of the DMC production process

In this section, the simulation of the DMC production process has been presented and the results have been compared with experimental data reported by Cui et al.^16^.

### Experiments

Cui et al. examined the reactions in the presence of a mixture of KI and K_2_CO_3_ with a mass ratio of (1:1) as a catalyst in a batch reactor. The feed was injected into the reactor then pressurized by injecting CO_2_ and heated to the desired temperature. At each experiment, up to 50 ml of 10% (wt.) Ethylene Oxide-methanol solution was injected into the reactor; raw materials and catalyst were mixed at 900 rpm. Finally, samples were taken from the reactor in the liquid phase at time intervals.

### Simulation of DMC production process

The DMC production process (the transesterification of ethylene oxide with methanol) has been simulated based on a kinetic study carried out by Cui et al. 2004 by Aspen HYSYS 10. The NRTL equation of state has been implemented to predict the thermodynamic behavior of compounds. The reactions were carried out in the presence of KI and K_2_CO_3_ as catalysts.

Simulation of the DMC production process has been performed in two stages. In the first stage, EO and CO2 reach to the reaction temperature and the first instantaneous reaction has occurred in a conversion reactor as follows^16^:

1 $$EO \left( {Ethylene Oxide} \right) + CO_{2} \to EC$$

The produced ethylene carbonate along with methanol injected into a CSTR and DMC is produced as follows (Cui et al. 2004) [23]:

2 $$EC + 2MEOH \to DMC + EG$$

3 $$r_{DMC} = \left[ {6.59*10^{2} *e^{{\frac{{ - 3.72*10^{4} }}{RT}}} *C_{EC}^{0.8} - 1.19*10^{4} *e^{{\frac{{ - 5.37*10^{4} }}{RT}}} *C_{DMC} C_{EG} } \right]$$

In addition; 2-Methoxyethanol (ME) is produced as a by-product as follows^16^:

4 $$EC + MEOH \to ME + CO_{2}$$

5 $$r_{ME} = 1.19*10^{6} e^{{\frac{{ - 8.24*10^{4} }}{RT}}} C_{EC}$$

**Figure 1:**
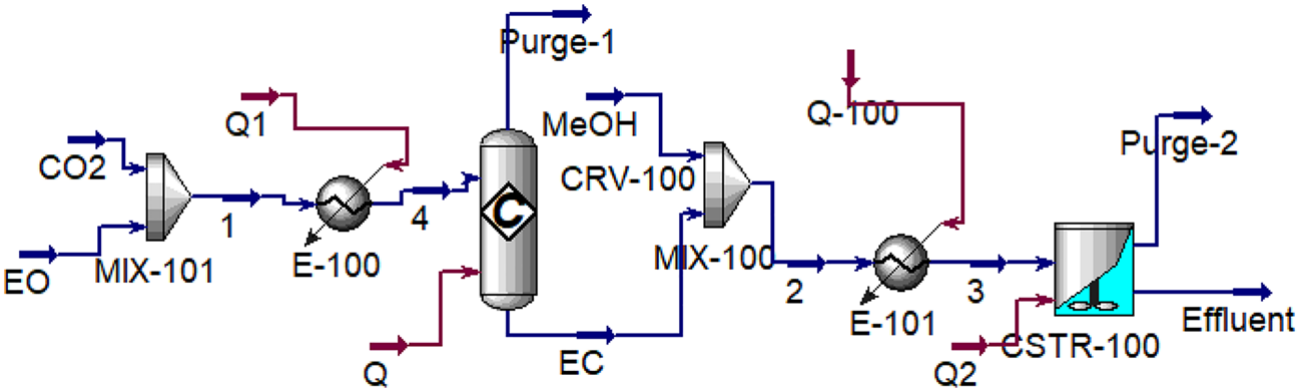
Simulation of DMC production process.

The simulation of the DMC production process in Aspen HYSYS has been shown in Fig. 1. The properties and conditions of feed have been presented in Table S1.

### Verifying the validity of results

**Figure 2:**
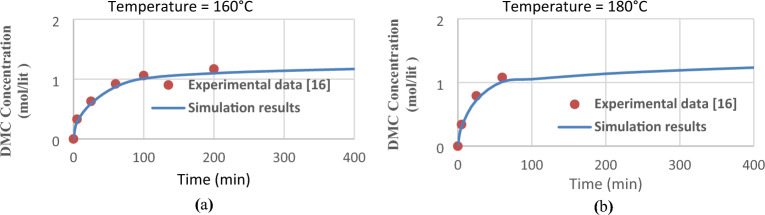
Comparison of experimental data and simulation results (**a**) 160 °C and (**b**) 180 °C.

Figure 2 shows the effect of residence time on DMC production at 160 °C and 180 °C for experimental data (Cui et al. 2004)^16^ and simulation results. Comparison of the simulation results with experimental data (Fig. 2) illustrates that the simulation results are in good agreement with experimental data. The average error 160 °C and 180 °C are 3.56% and 3.99%, respectively. So we can extend the simulation for investigating the effective parameters and optimizing the process.

### Developing the process

**Figure 3:**
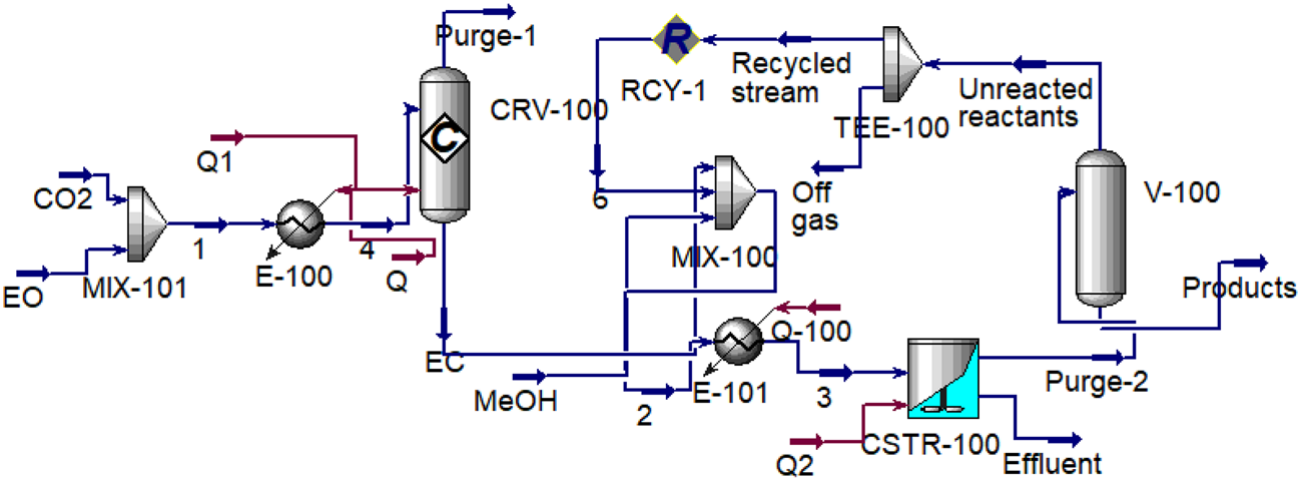
Simulation of modified DMC production process.

According to the accuracy of the simulation results, it has been used for developing the process for evaluating the effects of parameters and optimizing the system. For this purpose, a part of the unreacted reactants has been separated using a flash drum and recycled into CSTR. Figure 3 shows the developed process containing the flash drum and recycle stream.

### Process optimization

In this study, optimization of EC conversion has been performed by response surface methodology (RSM). In statics, RSM examines the interactions between a number of explanatory factors and one or more response variables. Sequential statistics of four-level Box-Behnken design using Design Expert 12 have been implemented to optimize the production of dimethyl carbonate. The significant parameters of the process including Temperature, recycle ratio, residence time, and MeOH/EO have been examined on EC conversion. Total of thirty runs of experiments have been carried out in a standard manner (Table S2). The values for the factor’s level for RSM analysis are as follows:

$$\begin{gathered} {135 } < {\text{A}}:{\text{ Temperature}}\left( {^\circ {\text{C}}} \right) < {165} \hfill \\ 0.{2}00 \, < {\text{B}}:{\text{Recycle ratio}} < 0.{6}00 \hfill \\ {75}.00 \, < {\text{C}}:{\text{Residence time }}\left( {{\text{min}}} \right) \, < { 125}.00 \hfill \\ {3}.{5}0 \, < {\text{D}}:{\text{MeOH}}/{\text{EO }} < { 8}.{5}0 \hfill \\ \end{gathered}$$

**Figure 4:**
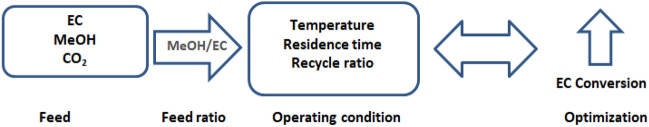
optimization algorithm.

Figure 4 shows the algorithm of optimization for DMC production process.

The quadratic VS 2FI model has been implemented to predict the response function and estimate the coefficients by multiple regression analysis^17^:

6 $$Y = \beta_{0} + \mathop \sum \limits_{j = 1}^{k} \beta_{j} X_{i} + \mathop \sum \limits_{j = 1}^{k} \beta_{ii} X_{i}^{2} + \mathop \sum \limits_{i}^{k - 1} \beta_{ij} X_{j} X_{i} + e$$

where Y is the predicted response, X_i_ and X_j_ represent the independent variables, β_0_, Β_ii_, β_ij_ are constant coefficients. k indicates the number of studied factors and e is the error value. Analysis of variance (ANOVA) has been implemented for model verification.

## Results and discussion

In this section, effective parameters in the DMC production process using carbon dioxide and ethylene oxide contains including temperature, feed ratio, and recycle ratio have been investigated.

### Effects of temperature on DMC production

**Figure 5:**
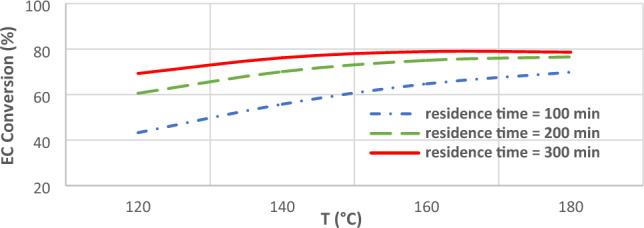
Effects of temperature on EC conversion in different residence times.

Figure 5 shows the effects of temperature on EC conversion in different residence times. DMC production process using carbon dioxide and ethylene oxide contains three reactions of Eqs. (1), (2), and (4). It is evident that increasing temperature enhances the conversion of ethylene carbonate. The effect of temperature on increasing the EC conversion in lower residence times is more significant compared to higher residence times. i.e., increasing the temperature from 120 ͦ C to 140 ͦ C increases the EC conversion from 43.22% to 55.74% in residence time of 100 min, while this value for residence time of 300 min varies from 69.25% to 76.16%. The mentioned trends have been also reported by Song et al. 2011^18^.

### The effect of feed ratio on EC conversion

**Figure 6:**
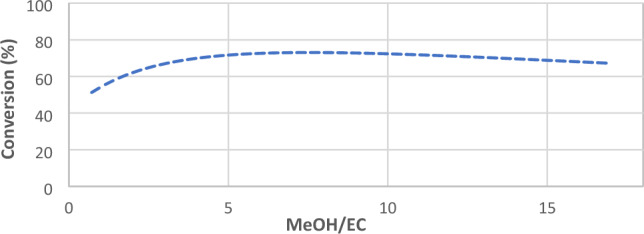
Effect of feed ratio (MeOH/EO) on EC conversion.

The feed ratio is one of the most influential factors in ethylene carbonate conversion. As mentioned, the first reaction (converting ethylene oxide to ethylene carbonate) is an instantaneous reaction, hence; the ratio of methanol (MeOH) to ethylene carbonate has been considered as an operating parameter. Figure 6 shows the effect of the MeOH/EC mass ratio on EC conversion. It is shown that a mild maximum point has occurred in the range of MeOH/EC = 6. This trend has been also observed by Cui et al.^16^.

### Effects of recycle ratio on EC conversion

**Figure 7:**
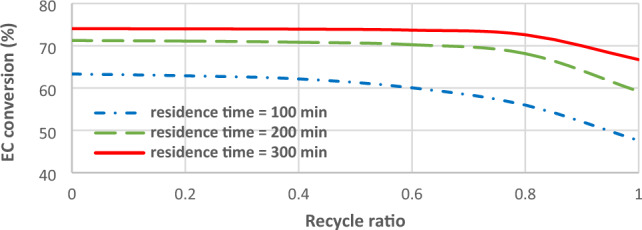
The effect of recycle ratio on EC conversion in residence times of 100, 200 and 300 min.

Figure 7 illustrates the effects of recycle ratio on EC conversion in different residence times. Recycling the unreacted reactants into the rector avoids wasting the feed and decreases the cost of the separation unit, however; reduces the residence time of reactants in constant reactor volume and disturbs the optimum feed\ ratio. A similar trend is shown at higher residence time, but the impact of recycle ratio on EC conversion is delayed up to 0.75–0.8.

### Analyses of variance (ANOVA)

In order to assess the interaction between effective parameters for obtaining an optimized range of EC conversion, Design Expert 12 has been used. The process has been studied using the quadratic VS 2FI model and square root transformation. The developed regression model’s sufficiently and validity have been evaluated using the ANOVA approach (Table S3). The analysis showed that linear, reciprocal, and square for the production of DMC are significant, with the exception of the interactions between recycle ratio-Feed ratio (BD) and residence time-feed ratio (CD). The Model F-value of 52.51 and P-values of less than 0.0001 imply the model is significant.

The adjusted R^2^ of 0.9464 and the predicted R^2^ of 0.9167 are reasonably in agreement. An ideal signal-to-noise ratio is more than 4, as measured by the Adeq Precision. The ratio of 29.525 indicates a sufficient signal, and the following parameter combinations can be used with this model to predict the responses:

7 $$Sqrt\left( {Conv} \right) = \, \left( \begin{gathered} 7.15 + 0.2866 \, A - 0.2795 \, B + 0.2703 \, C + 0.3126 \, D \hfill \\ \quad - 0.0738 \, AB - 0.0811 \, AC - 0.0043 \, BD \, \hfill \\ \quad - 0.0695 \, A - 0.1166 \, B - 0.0488 \, C - 0.0511 \, D \hfill \\ \end{gathered} \right)$$

**Figure 8:**
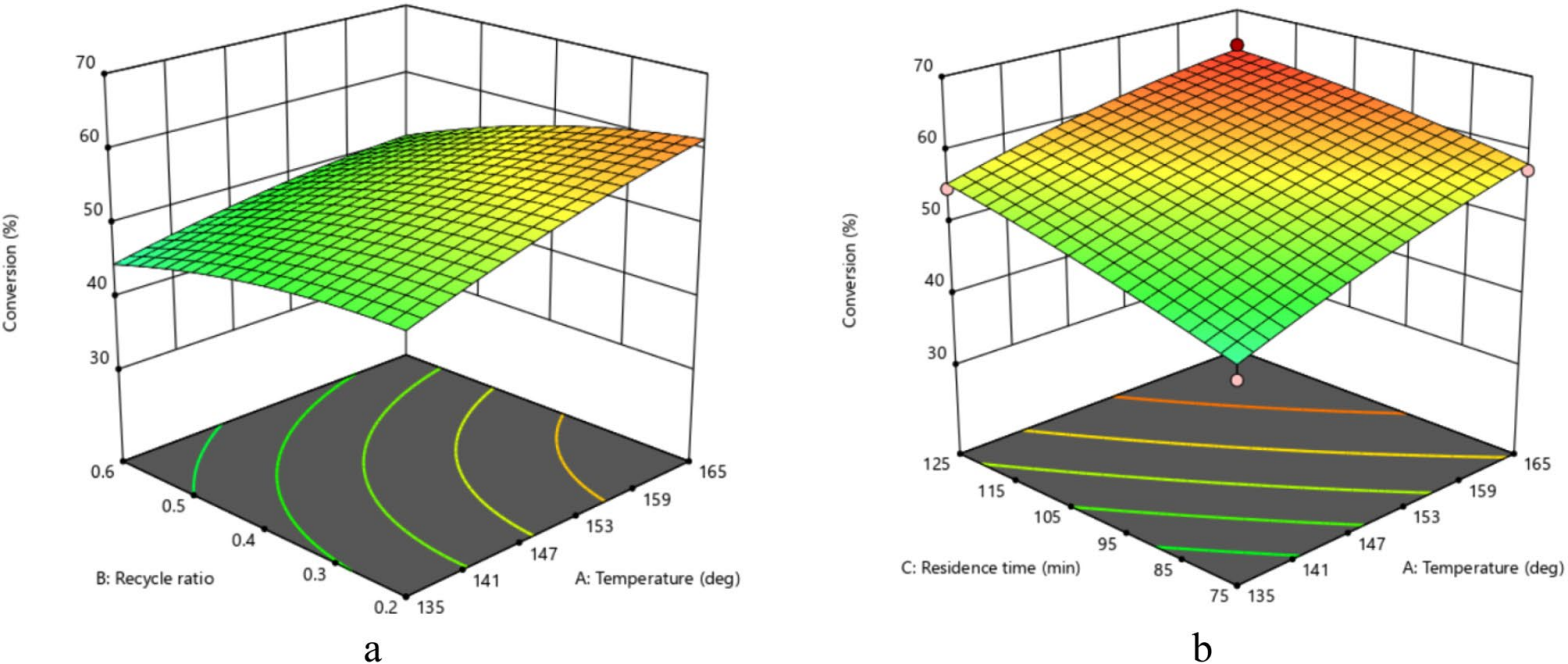
The effect of (**a**) temperature and recycle ratio interaction on EC conversion (residence time = 99 min and MeOH/EO = 8.5) (**b**) temperature and residence time interaction on EC conversion (Recycle ratio = 0.2 and MeOH/EO = 8.5).

Figure 8 shows the response surface diagram for temperature-recycle ratio and temperature-residence time interactions on the EC conversion. According to the ANOVA analysis, the interactions between temperature-recycle ratio and temperature-residence time have significant impacts on EC conversion. It was shown in Fig. 7 that increasing temperature and residence time simultaneously enhances the conversion of EC, However; recycle ratio had mild effects compared to the temperature.

## Optimization

In order to optimize the process and specify the best conditions to achieve proper DMC production, constraints have been applied to parameters according to the allowed range. Table S4 shows Process effective parameters and their applied constrains. Several combinations of parameters reported by Design Expert as optimum values have been shown in Table 1. According to Fig. 5 to Fig. 8 along with Table 1, the recycle ratio < 0.7 has not significant effects on EC conversion, however; significantly increases the cost of separation unit due to the existence of azeotrope between methanol and DMC and challenges of separation of homogeneous catalyst. Increasing MeOH/EC increases the conversion of reversible reaction of DMC production (Eq. 2). In contrast, increasing the recycle ratio and MeOH/EC increase reactor volume. The reaction temperature is low and does not significantly affect the cost of the process. So, the best set of the reported optimum conditions is selected as raw 1.

**Table 1:** Optimum combination of parameters for DMC production.

No	Temperature (°C)	Recycle ratio	Residence time (min)	MeOH/EO	Conversion (%)
1	164.7	0.201	139.45	5.9	70.12
2	160.1	0.199	141.18	4.1	65.47
3	136.2	0.800	145.94	8.5	63.60

## Conclusion

In this study, CO_2_ utilization for DMC production as a green and environmental friend process has been simulated and optimized. For this purpose simulation of the process has been performed by Aspen HYSYS 10 and operating conditions of temperature, residence time, feed ratio, and recycle ratio have been evaluated. It is shown that increasing the temperature and residence time increases the conversion of EC. Ethylene carbonate conversion versus MEOH/EC has a mild maximum. In addition; optimization of the process has provided several combinations of parameters as optimum points and temperature of 164.7 ͦ C, Recycle ratio of 0.2, residence time of 139.45 min and MeOH/EC of 5.9 have been selected as optimum operating condition of process which results in 70.1% for EC conversion.

### Supplementary Information

Supplementary Information 1.

Supplementary Information 2.

Supplementary Information 3.

Supplementary Information 4.

## Data Availability

All data generated or analysed during this study are included in this published article [and its supplementary information files].
