# Supplementary material for: Simulation and multi-objective optimization of the dimethyl carbonate production process
Source: Sci Rep. 2023 Oct 6;13:16900. doi: 10.1038/s41598-023-44100-y (PMC10558474; doi:10.1038/s41598-023-44100-y)
Supplement: Supplementary file 1 — Supplementary Information 1. [file 41598_2023_44100_MOESM1_ESM.docx]

**Table S1: the properties and conditions of feed [16].**

| Compound/Condition | CO_2_ | EO | EC | MET |
| --- | --- | --- | --- | --- |
| Temperature/ °C | 10 | 171 | 140 | 211.6 |
| Pressure/KPa | 5000 | 5000 | 5000 | 5000 |
| Flow/mol | 397.6 | 397.2 | 397.6 | 764.6 |
| CO_2_ | 1 | 0.001 | 0 | 0 |
| EO | 0 | 0.999 | 0 | 0 |
| EC | 0 | 0 | 1 | 0 |
