# Supplementary material for: Simulation and multi-objective optimization of the dimethyl carbonate production process
Source: Sci Rep. 2023 Oct 6;13:16900. doi: 10.1038/s41598-023-44100-y (PMC10558474; doi:10.1038/s41598-023-44100-y)
Supplement: Supplementary file 2 — Supplementary Information 2. [file 41598_2023_44100_MOESM2_ESM.docx]

**Table S2: Values of response levels for EC conversion**

| Std | Run | Factor 1  A: Temperature  (°C) | Factor 2  B: Recycle ratio | Factor 3  C: Residence time (min) | Factor 4  D:(MEOH/EO) | Response  Conversion (%) |
| --- | --- | --- | --- | --- | --- | --- |
| 1 | 2 | 135 | 0.2 | 75 | 3.5 | 37.8245 |
| 2 | 24 | 165 | 0.2 | 75 | 3.5 | 48.1903 |
| 3 | 1 | 135 | 0.6 | 75 | 3.5 | 35.5265 |
| 4 | 4 | 165 | 0.6 | 75 | 3.5 | 41.4923 |
| 5 | 26 | 135 | 0.2 | 125 | 3.5 | 47.3651 |
| 6 | 3 | 165 | 0.2 | 125 | 3.5 | 53.6671 |
| 7 | 29 | 135 | 0.6 | 125 | 3.5 | 42.1322 |
| 8 | 23 | 165 | 0.6 | 125 | 3.5 | 44.5734 |
| 9 | 18 | 135 | 0.2 | 75 | 8.5 | 43.5476 |
| 10 | 6 | 165 | 0.2 | 75 | 8.5 | 57.342 |
| 11 | 14 | 135 | 0.6 | 75 | 8.5 | 40.5541 |
| 12 | 5 | 165 | 0.6 | 75 | 8.5 | 49.4905 |
| 13 | 21 | 135 | 0.2 | 125 | 8.5 | 54.8118 |
| 14 | 17 | 165 | 0.2 | 125 | 8.5 | 64.7839 |
| 15 | 7 | 135 | 0.6 | 125 | 8.5 | 49.5646 |
| 16 | 8 | 165 | 0.6 | 125 | 8.5 | 54.4772 |
| 17 | 15 | 120 | 0.4 | 100 | 6 | 38.7781 |
| 18 | 13 | 180 | 0.4 | 100 | 6 | 54.8012 |
| 19 | 27 | 150 | 0 | 100 | 6 | 55.0721 |
| 20 | 22 | 150 | 0.8 | 100 | 6 | 34.0122 |
| 21 | 20 | 150 | 0.4 | 50 | 6 | 39.9588 |
| 22 | 10 | 150 | 0.4 | 150 | 6 | 55.8651 |
| 23 | 30 | 150 | 0.4 | 100 | 1 | 37.9203 |
| 24 | 28 | 150 | 0.4 | 100 | 11 | 58.0576 |
| 25 | 16 | 150 | 0.4 | 100 | 6 | 51.1445 |
| 26 | 25 | 150 | 0.4 | 100 | 6 | 51.1445 |
| 27 | 12 | 150 | 0.4 | 100 | 6 | 51.1445 |
| 28 | 9 | 150 | 0.4 | 100 | 6 | 51.1445 |
| 29 | 19 | 150 | 0.4 | 100 | 6 | 51.1445 |
| 30 | 11 | 150 | 0.4 | 100 | 6 | 51.1445 |
