# Supplementary material for: Simulation and multi-objective optimization of the dimethyl carbonate production process
Source: Sci Rep. 2023 Oct 6;13:16900. doi: 10.1038/s41598-023-44100-y (PMC10558474; doi:10.1038/s41598-023-44100-y)
Supplement: Supplementary file 3 — Supplementary Information 3. [file 41598_2023_44100_MOESM3_ESM.docx]

**Table S3: Analyses Of Variance (ANOVA)**

| **Source** | **Sum of Squares** | **df** | **Mean Square** | **F-value** | **p-value** |  |
| --- | --- | --- | --- | --- | --- | --- |
| **Model** | 8.62 | 10 | 0.8625 | 52.21 | < 0.0001 | significant |
| A- temperature | 1.97 | 1 | 1.97 | 119.34 | < 0.0001 |  |
| B- recycle | 1.88 | 1 | 1.88 | 113.51 | < 0.0001 |  |
| C- residence time | 1.75 | 1 | 1.75 | 106.10 | < 0.0001 |  |
| D-MEOH/EO | 2.35 | 1 | 2.35 | 141.94 | < 0.0001 |  |
| AB | 0.0871 | 1 | 0.0871 | 5.27 | 0.0333 |  |
| AC | 0.1053 | 1 | 0.1053 | 6.37 | 0.0206 |  |
| A² | 0.1325 | 1 | 0.1325 | 8.02 | 0.0107 |  |
| B² | 0.3730 | 1 | 0.3730 | 22.58 | 0.0001 |  |
| C² | 0.0653 | 1 | 0.0653 | 3.95 | 0.0614 |  |
| D² | 0.0715 | 1 | 0.0715 | 4.33 | 0.0513 |  |
| **Residual** | 0.3139 | 19 | 0.0165 |  |  |  |
| Lack of Fit | 0.3139 | 14 | 0.0224 |  |  |  |
| Pure Error | 0.0000 | 5 | 0.0000 |  |  |  |
| **Cor Total** | 8.94 | 29 |  |  |  |  |
| **R²** | 0.9649 |  |  |  |  |  |
| **Adjusted R²** | 0.9464 |  |  |  |  |  |
| **Predicted R²** | 0.9167 |  |  |  |  |  |
| **Adeq Precision** | 29.5250 |  |  |  |  |  |
