# Supplementary material for: Simulation and multi-objective optimization of the dimethyl carbonate production process
Source: Sci Rep. 2023 Oct 6;13:16900. doi: 10.1038/s41598-023-44100-y (PMC10558474; doi:10.1038/s41598-023-44100-y)
Supplement: Supplementary file 4 — Supplementary Information 4. [file 41598_2023_44100_MOESM4_ESM.docx]

**Table S4: Process effective parameters and their applied constrains**

| **Name** | **Goal** | **Lower Limit** | **Upper Limit** | **Lower Weight** | **Upper Weight** | **Importance** |
| --- | --- | --- | --- | --- | --- | --- |
| A: Temperature  (°C) | is in range | 135 | 165 | 1 | 0.1 | 3 |
| B: Recycle ratio | maximize | 0.2 | 0.8 | 10 | 1 | 4 |
| C: Residence time (min) | minimize | 50 | 150 | 1 | 0.1 | 4 |
| D: MEOH/EO | is in range | 3.5 | 8.5 | 1 | 1 | 3 |
| Conversion (%) | maximize | 34.0122 | 64.7839 | 3.98107 | 1 | 5 |
